# 

**Published:** 1842-09

**Authors:** 


					THE
AMERICAN JOURNAL
OF
DENTAL SCIENCE.
THE
AMERICAN JOURNAL
OF
Dental Suicnce.
PUBLISHED UNDER THE AUSPICES OP THE
American <Soctet2> of Dental Surgeons.
VOLUME III.?1842-3.
editors:
CHAPIN A. HARRIS, M. D.
SOLYMAN BROWN, M.D.
LEONARD MACKALL, M. D.
NEW YORK:?GEORGE ADLARD,
BALTIMOR E ARMSTRONG & BERRY,
LONDON :?SAMUEL. HIGHLEY.
1843.
w
i
A/A
<V5
/A
WOODS & CRANE, PRINTERS,
Baltimore.
INDEX.
PAGE.
Address, Introductory, by E.
Parmly, M. D., . . . 1-75
American Society of Dental Sur-
geons,  69
Artificial teeth worn by Wash-
ington, . . . .76
Amputation of the lower jaw,
by C. B. Gibson, M. D., . 138
Anonymous communications, . 148
Anomalous tooth, . . 150
Abscess, Intra-dental, . 278-281
Animale Regnum, * . 286
Anniversary, Fourth, of Ameri-
Society of Dental Surgeons, 290
Another tooth injected with red
blood, .... 292
B
Boylston prize question, . .78
Brown, S., M. D., D. D. S., on
mechanical dentistry, 81-189-233
Brewster, C. S., M. D., D. D. S. 149
Baltimore College of Dental Sur-
gery,  151
British Quarterly Journal of Den-
tal Surgery, . . . 288
Catalogue of officers and mem-
bers of American Society of
Dental Surgeons, .
Correspondents, to,
Calculus, Essay on, .
Contributions to dentistry, by
W, H. Elliot, .
PAGE.
Communications, Anonymous, 148
College of Dental Surgery, Bal-
timore, .... 151
Comments, &c. by Dr. Hayden,
203-246-286
Convulsions, lancing the gums
in, 228
Cellular structure of the teeth,
Memoir on, . . , . 229
Clark, Dr. C. J., letter from, 277
D.
Dissertation on the Maxillary
Sinus, by C. A. Harris, M. D.,
D. D. S., . . . 20-98-153
Dental Surgeons, American So-
ciety of, . . . .69
Dentistry, Mechanical,
77-81-189-233
Dissertation on degrading the
dental profession, , . .78
Drill Stock, Dr. Maynard's 80-228
Dentistry, Contributions to, . 133
Dentists, Practice of, . 144-145
Dentists, Virginia Society of, . 145
Dentists, Convention of . . 146
Deciduous Teeth, osseous union
of, 150
Dental Surgery, Baltimore Col-
lege of, ... 151
Development, structure and dis-
eases of the teeth, . . .73
Dental Art, by Maury, . . 226
Dictionary of Practical Surgery, 285
Dental Surgeons, fourth anni-
versary of, .... 290
6 INDEX.
PAGE.
Dental Surgery, practice of, . 291
E.
Elliot,, W. H., method of pivot-
ing teeth, .... 80
Elliot, W. H., contributions to
dentistry,. . . .? .133
Extracts from Proceedings of
Convention of Dentists, . 146
Extraordinary case of diseased
gums, .... 240
Extraction of teeth, observations
on, 286
Errata in Dr. Hay den's Com-
ments, .... 292
F.
Formation of the ivory of the
teeth, .... 230
Fourth anniversary of the Am.
Society of Dental Surgeons, 290
G.
Gunnel, J. S., M. D., on protru-
sion of the lower jaw, . 65
Greenwood, Dr. Wm. P., and
Mr. Peabody, . . .77
Gums, lancing of, . . 228
Gums, fungous disease of, . 240
Greenwood, I. I., M. D., on
plugging teeth, . . . 237
Grimes, Samuel, on the treat-
ment of intra-dental abscess, 279
H.
Harris, Chapin A., M. D., Dis-
sertation on the Maxillary Si-
nus, .... 20-98-153
Hayden, A. B. obituary notice of, 80
Handbuch's Dental Surgery . 144
Honours, more, . . .150
Hay den, H. H. Comments,
203-246-286
I.
Introductory Address. By E.
Parmly, M. D., D. D. 1-75
Intra-dental Abscess, . 278-281
PAGE.
J.
Jaw, lower, amputation of, . 138
Journal of Dental Surgery, Brit-
ish Quarterly, . . . 282
K.
Koecker, L., M. D., D. D. S.a
upon the diseases of the gums, 240
L.
Lancing the gums, . . . 228
Lectures, spring course of, in
College of Dental Surgery, . 151
Lefoulon, J., Part du dentiste, 144
Likeness, Dr. E. Parmly's, . 149
Linderer, Von, C. J. Dental Sur-
gery,  144
M.
Maxillary Sinus, diseases of,
20-98-153-203-246
Mechanical Dentistry,
77-81-133-189-233
Medical Prize Question, . . 78
Method of degrading the dental
profession, . . . .78
Maynard's, Dr. Drill Stock, 80-228
Maxillary Bone, excision of, . 142
Maury's Dental Surgery, 145-226
Minutes of Richmond Dental
Convention, . . . 146-227
Mania from decayed teeth, . 152
More honours, ./ . . .150
Memoir upon the cellular struc-
ture of the teeth, . . 229.
N.
Notice from Treasurer, . . 78
Nasmyth, A., M. R. C. S. L.,
upon the structure of the teeth
and pulps, .... 229
o.
Officers and members, catalogue
of, 73
Operative and mechanical Den-
tistry,  133
INDEX.
PAGE.
Obituary, A. B, Hayden, . 80
Osteo-Sarcoma of lower jaw, by
C. B. Gibson, M. D. . . 138
Our reprint, . . . .149
Osseous Union of Deciduous
Teeth, . . . .150
Odontology, some points upon, 229
Obturators and palates, artificial, 233
Observations on extraction of
teeth, ..... 286
Omission, \ 292
Obituary, 292
p.
Parmly, E., M. D., D. D. S.,
Address of, . . . .1
Protrusion of lower jaw, remedy
. for, 65
Peabody and W. P. Greenwood, 77
Pivoting teeth, method of, . 80
Pratique et Theorique de Part du
dentiste, . . . .144
Parmly, E. likeness of, . . 149
Proceedings and minutes of Vir-
ginia Society of Dental Sur-
geons,  227
Plugging teeth, by I. I. Green-
wood, M. D., . . . 237
Practical Surgery, a dictionary of, 285
o,
Questions, Prize, Medical, . 78
Quarterly Journal of Dental Sur-
gery, The British, . . . 282
R.
Remedy for protrusion of lower
jaw, . . .
Richmond Convention of Dental
Surgeons, ....
Reprint, ....
Remedies for tooth-ache, .
Rostang, Dr. A. .
s.
Sinus, Maxillary, diseases of, 20-153
Society, of Dental Surgeons,
American, . . . .09
Society of Surgeon Dentists,
Virginia, . . . .145
paoe.
Surgeon Dentists, a convention
of, . . . . .146
Spring Lectures of Baltimore
College of Dental Surgery. . 151
Society of Dental Surgeons, wes-
tern, formation of, . . 152
Sinus, Maxillary, essay on, . 203
Stridulous Covulsions, . . 228
Structure of the Teeth and their
Pulps, ..... 289
Saunders, Edward, on intra-
dental abscess, . . .278
Surgery, Practical, a Dictionary
of, 285
T.
Tooth bone, vascularity of, .78
Treasurer, notice from, . . 80
Teeth, pivoting, method of, . 80
Tooth, Anomalous, . . 150
Teeth, deciduous, osseous union
of, . . . . .150
Tooth-ache, remedies for, . 151
Teeth, decayed, mania from, . 152
Taylor, Dr. James, . . 152
Treatise on Mechanical Dentis-
try, - . . . . .189
Treatise on the Dental Art, . 226
Teeth, structure of, . . . 229
Teeth, on plugging the, . 237
Teeth, extraction of, . . 286
u.
Upper Maxillary Bone, Excision
of, 142
V.
Vascularity of tooth bone, . 78
Virginia Society of Surgeon Den-
Mists,  145
Vent in the stopping of a decayed
tooth, .... 278-281
w.
Washington, artificial teeth worn
by, 76
Western Society of Dental Sur-
geons,  152
To Correspondents.?We wish our correspondents to bear in mind that the
editors of the American J ournal and Library of Dental Science are directed,
by a special resolution of the American Society of Dental Surgeons, as are
also the officers of said association, not to take any letter from the post-office,
relating to the society or Journal, on which the postage has not been paid,
except they contain money, or come from abroad. The item of postage has
heretofore been so considerable, that the society deemed the adoption of a
resolution of this kind necessary.
While upon this subject, we would suggest to our agents and subscribers
that in making remittances for the Journal, they may save both themselves
and the society the expense of postage, by availing themselves of a regula-
tion of the post-office department, by which post-masters are permitted to
frank letters, when written by themselves, containing money, to pay sub-
scriptions to newspapers or periodicals.
The following gentlemen were elected at the last meeting of "
Surgeons, Collaborators of the American Journal add Library of
H. H. HAYDEN, M. D.
ELEAZAR PARMtY, M. ft
EDWARD MAYNARD, M. D.
H. .M. HAYDEN, M. D.
ELISH A TOWNSEND,
VERNON CTTYLOR, M. D.
Hartford, Ct.
ISAAC I.
G. G. BREWSTER,
LUDOJLPH PARMLY,M D.
b Ala.
EDWARD TAYLOR, M. D.
z, SB
C. M BRE WSTJEE, M B.
Pari*, France.
LEONARD KOECKER, M. D.
London,
LEWIS ROP?R, M. D.
Philadelphia.
H. S. BUHR, M. D.
Philadelphia
E. BAKER, M. 0.
u
'B. A. R0BRI6UES, M. D.
ROGERS, M.
ENOCH NOYES, M. ?.
Baltunoie.
SUBSCRIPTIONS TO THE
ence to the Prospectus on the fourth page of the cover, that the terms of subscription are payment
in advance. The Journal having become the property of the 1
geons, the conductors of the publication are required by that body, to see that this condition is
strictly complied with ; and, they mention it, that those of the
have signified their wish to continue their subscription, without Saving jfeidfhe amount, may nof
attach any blame to the
OFFICERS AND MEMBERS OF Tl
SURGEONS.?A catalogue of the
lished by the Recording Secretary, by order of the ss
in a convenient form for framing.
curea by application to the above named officer, on parchment, silk, paper or white satm,
'vy.
-The
nished with the same, by transmitting ten
?       M .
to the Second Volume of the American Journ
and Library of Dental Science.
,er, twelve subscriptions........  #60 00
500
-????-!  ???l
Dr. Gilbert,. .....T............y? **&&&; 5 0?
!)<??>?? >??? ? < t ? ? >?<???<? )?? > >i ? ???? 5 00
\ Savipr
s. oaviqr,.^    .?
'Donald,, ? *?? ?     ? ? ? ??? > ? #?? ? ? ? ? ? ? ? ? ?
?&& iBf - st if ^ ST - ??*? .. VOhJ. "
r
Illott,
8
]a \ * ??
Ie>   ?
>????<? ? v ? ? ? ? ........ ? ? .......
M. S< Foster,     5 00
T o??a WQ&Smssi-'* ftft
j . rvees,   .............. ?-? #>. o uu
?s mm, :,r
J. F. Cassell,. ...,....... * *....... ?.. ?| ?/... $ 5 00
in, v,.
Dr. H. N;F?m  y.........,,v.......y 'i 5 00
W. Johnson, Hagarstown, Md.   .  5 00
5
?????*??????????????????????????? 5 Od
- , "1. D. Hartford, Conn  6 00
APPLICATIONS FOE MEMBERSHIP.
During the last session of the American Society of Dental Surgeons. Commit-
tees were appointed in the cities ot Boston, JNew York. Philadelphia, and Baltimore,
to receive the applications or those who wished to be presented at the
Meeting of the Society for membership.
Those wishing , to become members; will please apply as above to
members of the Committees, whose names they will find in the proceedings, publish-
ed u
EDITORS' PROSPECTUS
OP THE
v
As this Journal has become the organ of the American Society of Dental *?
nam? has been somewhat modified, it may be considered as having entered upon a new period of
its existence; and its former publishers congratulate themselves and the profession o
fortune. Its perpetuity is now considered as settled. The
devoted to its interests, and who is better able to conduct it, than the only
extensive association of dental surgeons in the world. We repeat, that we congratulate ourselves!
and our professional brethren on an event so auspicious to the science of dental surgery, as the
formation of a national society and the transfer of the journal to Jhat body. The work can now bag)
regularly issued, whether its circulation be co-extensive with the profession or not. If the subscrip- f
tion should be limited, the issues can be made to correspond with the demand, so that the exper
to the society may be kept wholly within its means.
The American Journal and Library of Dental Science, is now proposed to take an
ground, and will hereafter solicit no favours from those members of the profession who are opposed
to the dissemination of periodical information. To others who are favourably inclined to ^a comir
nity of knowledge, this journal will not fail to commend itself, as one of the most efficacious means
of exposing the impositions of quackery, and imparting to
mation which their usefulness and talents should secure.
The journal will be regularly issued on the first of December, March, June and September, 1
annually, at the enhanced price of five dollars per volume, payable in all cases in advance. The
price of subscription may be paid to the treasurer of the society, Dr. Leonard
city, or to either of the editors, or any of the publishers, collaborators or acknow
the journal. Advance payment is required by a special resolution, passed at the last i
society, and is necessary to protect it from pecuniary loss, a neglect of which
publishers of the first volume in unexpected embarrassment, if not in ultimate loss.
To save postage, the agents in all the large cities of the United States, and Great
furnished witfc copies to supply subscribers, by such conveyance as they shall prescribe. And
where it can be done, even, individuals may receive their numbers by private conveyance if theyl
choose.
With these few explanatory remarks, we commit our work to its coming destiny,
the profession that no efforts will be spared to render it every way worthy of the great subject to
which its pages will be exclusively devoted.
CHAPIST a. HARRIS,
SOLYMAN
LEONARD MAC?A3
Besides the Collaborators, the following gentlemen are regularly
t*v 1 ' 7'-i "'? l/> ">?' >WV.-;J Viki -;'i -v ' * "" ' ' >*- v' U* jWr.
AGENTS OF THIS JO""" * *
Bradbury & So dew,... .... .Boston.
F. B. Chewning, Richmond, Va.
J. P. Laird, M. D..     .Columbus, Ga.
Geo. Washington Pahmly, New Orleans.
G. McDonald, M. D ....... IVfacon, Ga.
Blandin & Avery, ........Columbia, S. C.
Eleazae Gidney, Manchester, Eng.
J. Allen,   Cincinnati, Ohio.
A. B. Hayden, D.D. S Savannah, Geo.
Dbs. Goddard and Ferguson, Louisville, Ky.
John Harris, M.D........ ..G
Nicholas Clots,, ......,.. Xc
J. N. Frink
** ? ? ? >
............... DU
Benj. Strickland,
B. B. Brown, M. D . m. St. Louis,
V
W, R. Scott, D. D. S  Rakigh, N, C.
S. M. Shepherd,. 4 ........ .Petersburg, Va. '
W. W. H Thackston, D.EL.S. Farmville, Va.
Edward Taylor, M. D, ... .Natchez, Miss.

				

